# Intrinsic terminators in *Mycoplasma hyopneumoniae* transcription

**DOI:** 10.1186/s12864-015-1468-6

**Published:** 2015-04-08

**Authors:** Tiago Ebert Fritsch, Franciele Maboni Siqueira, Irene Silveira Schrank

**Affiliations:** Centro de Biotecnologia, Programa de Pós-Graduação em Biologia Celular e Molecular, Universidade Federal do Rio Grande do Sul (UFRGS), Porto Alegre, RS Brazil; Departamento de Biologia Molecular e Biotecnologia, Centro de Biotecnologia, Universidade Federal do Rio Grande do Sul (UFRGS), Av. Bento Gonçalves 9500, P. 43421, C.P. 15005, Porto Alegre, RS CEP 91501-970 Brazil

**Keywords:** *Mycoplasma hyopneumoniae*, Transcription termination, Intrinsic terminator, Transcriptional units

## Abstract

**Background:**

*Mycoplasma hyopneumoniae*, an important pathogen of swine, exhibits a low guanine and cytosine (GC) content genome. *M. hyopneumoniae* genome is organised in long transcriptional units and promoter sequences have been mapped upstream of all transcription units. These analysis provided insights into the gene organisation and transcription initiation at the genome scale. However, the presence of transcriptional terminator sequences in the *M. hyopneumoniae* genome is poorly understood.

**Results:**

*In silico* analyses demonstrated the presence of putative terminators in 82% of the 33 monocistronic units (mCs) and in 74% of the 116 polycistronic units (pCs) considering different classes of terminators. The functional activity of 23 intrinsic terminators was confirmed by RT-PCR and qPCR. Analysis of all terminators found by three software algorithms, combined with experimental results, allowed us to propose a pattern of RNA hairpin formation during the termination process and to predict the location of terminators in the *M. hyopneumoniae* genome sequence.

**Conclusions:**

The stem-loop structures of intrinsic terminators of mycoplasma diverge from the pattern of terminators found in other bacteria due the low content of guanine and cytosine. In *M. hyopneumoniae*, transcription can end after a transcriptional unit and before its terminator sequence and can also continue past the terminator sequence with RNA polymerases gradually releasing the RNA.

**Electronic supplementary material:**

The online version of this article (doi:10.1186/s12864-015-1468-6) contains supplementary material, which is available to authorized users.

## Background

Transcription is a highly regulated multi-step process roughly divided into initiation, elongation and termination. Prokaryotic transcription termination can occur via factor-dependent or factor-independent termination and is known to play key roles in regulating genetic systems. Factor-independent termination occurs at defined sequence regions known as intrinsic terminators and factor-dependent termination relies on the destabilisation of transcription complexes by a Rho regulatory protein [1]. Sequence features within intrinsic terminators have been studied in different organisms resulting in models for how these motifs contribute to overall termination efficiency, and have been used to develop computational methods to identify terminator elements within genome sequences [2-4].

Mycoplasmas are cell wall-less prokaryotes of the class Mollicutes, phylogenetically related to Gram-positive bacteria and characterised by having small genomes with a low GC content. *Mycoplasma hyopneumoniae* is considered the etiological agent of porcine enzootic pneumonia, a disease with global distribution and causing significant economic losses in the pig farming industry [5].

In recent years, the genomes of many *Mycoplasma* species have been completely sequenced, including those of some *M. hyopneumoniae* strains [6-8]. The sequencing of several mycoplasma genomes has provided an extensive comparative analysis of gene content among different species and information related to gene organisation in large transcriptional units (TUs) [8-10]. Moreover, promoter sequences have been defined and mapped for the *M. hyopneumoniae* genome [11,12] and recently, transcriptome analysis has validated the organization of *M. hyopneumoniae* genome in long transcriptional units [10].

However, despite the genome-scale sequencing efforts, prediction and recognition of mycoplasma terminator elements is poorly known. The unusually low GC content of intergenic regions (IRs) in the mycoplasma genome [13] may be the main reason for the lack of success in application of existing computational methods developed to predict putative intrinsic terminators. Moreover, gene coding for Rho regulatory protein was not found in mycoplasma genomes suggesting the absence of factor-dependent transcription termination in these organisms [4].

Studies investigating the presence of intrinsic terminators in mycoplasma were initially contradictory. Analysis of average RNA folding energy near stop codons demonstrated that no stem-loops were formed in *Mycoplasma genitalium* or *Mycoplasma pneumoniae* coding sequences (CDS), indicating the existence of qualitatively different and uncharacterised mechanisms for transcription termination [14]. However, the development of algorithms to predict terminators in low GC content genomes allowed the identification of stem-loop structures in some positions of mycoplasma genomes [3,15-17]. Furthermore, several studies showed that intrinsic terminators have a functional role in mycoplasmas [18-20] suggesting that the intrinsic terminators could be the main mode of termination.

Previously, we have described the genome organisation profile and mapped promoter sequences upstream of all transcription units of the *M. hyopneumoniae* genome [9,11,12]. These results provided insights into the gene organisation and transcription initiation at the genome scale. To further understand the mechanism of transcription in *M. hyopneumoniae* genome, in the current study we have analysed and predicted the presence of terminator sequences downstream of the transcription units. We have also determined the role of these predicted sequences, revealing that intrinsic terminators are the main mechanism of transcription termination in *M. hyopneumoniae*.

## Methods

### In silico analysis of terminators

**Figure 1:**
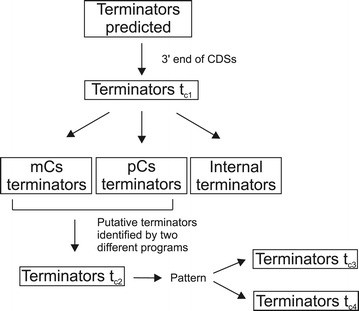
***In silico*** **design workflow utilised to classify terminators predicted in** ***M. hyopneumoniae*** **7448.** Predicted terminators were analysed by the software ARNold, TransTermHP and WebGesTer.

The prediction of terminator sequences was performed in *M. hyopneumoniae* 7448 (NC_007332) using three software algorithms: TransTermHP [16], WebGesTer [17] and ARNold [21]. The software WebGesTer restricts the search for palindrome sequences in a region ranging from −20 to +270 bp from the CDS stop codon and allows the search of non-canonical terminators (without the U-tract). TransTermHP algorithm restricts the search of palindrome sequences to regions in which occurs the presence of at least three thymines in sequence and, therefore, unable to find non-canonical terminators. ARNold combines two algorithms, Erpin and RNAmotif, which are based on terminators of *Escherichia coli* and *Bacillus subtilis* and do not consider the gene context in which the terminators occur. Figure 1 shows the workflow of our terminator prediction classification. Class 1 terminators (t_c1_) were defined using genome localisation parameters as follows: terminator sequences located at the 3’ end of the last gene of the polycistronic unit (pC); terminator sequences located at the 3’ end of the monocistronic unit (mC); terminator sequences located at the 3’ end of genes within the pCs. For subsequent analyses steps we have selected all terminator sequences located at the 3’ end of pCs or mCs and established new criteria for terminator classification: i) class 2 terminator (t_c2_) sequences were considered when positioned at the end of pC or mC and predicted by at least two different algorithms; ii) class 3 terminators (t_c3_) classification considered all t_c1_ with two additional features: first the distance between the terminator first nucleotide and the stop codon of the target gene should range from −11 to 200 base pairs and second, the values of Gibbs free energy (ΔG) should be less than −4 kcal/mol; and iii) class 4 terminators (t_c4_) were all t_c1_ that contained only one of the features of class 3 terminators. The terminators in the *M. hyopneumoniae* genome were mapped using *Artemis* software [22].

### Primer design

**Figure 2:**
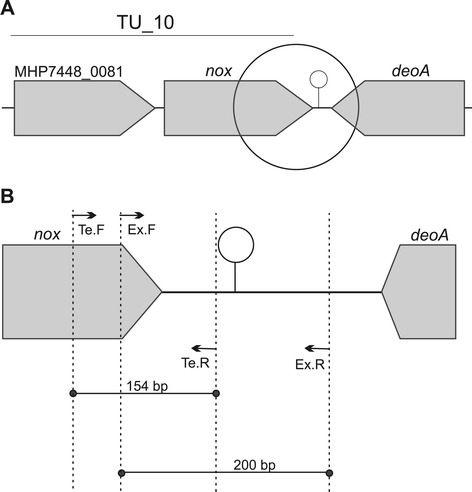
**Schematic representation of primers design for experimental analyses by RT-PCR or qPCR. A**: Representation of polycistronic unit 10 with the terminator w_014 (hairpin). **B**: Enlargement of highlighted region in A, showing the position of primers for terminator validation. Black arrows Te.F, Te.R, Ex.F and Ex.R represent the primers U.10.Te.F, U.10.Te.R, U.10.Ex.F and U.10.Ex.R, respectively. (U.10 – polycistronic unit 10, Te – terminator, Ex – external, F – forward and R – reverse).

Specific RT-PCR and qPCR primers were designed (based on the *M. hyopneumoniae* genome sequence — GenBank access: NC_007332) to target transcription before and after the selected predicted position of the terminators. Figure 2 illustrates an example (*M. hyopneumoniae* 7448 TU_10) of the position of primer pairs to validate the putative terminator. Figure 2A shows a global view of the putative terminator w_014 and Figure 2B demonstrates a detailed position of the primers with the product length expected. As shown in Figure 2B we designed two pairs of primers for each terminator analysis for both RT-PCR and qPCR methodologies. Briefly, the forward primers were positioned inside the coding region of the selected CDS (named Te.F and Ex.F or Ge.F when the primer forward was the same for the two reactions). One reverse primer was positioned between the stop codon of the CDS and the start of the putative terminator (upstream terminator – Te.R). The second reverse primer was positioned immediately after the putative terminator (downstream terminator – Ex.R). A total of 60 primer pairs were prepared for confirmation of 28 putative terminators (Additional file 1). Primers were designed in *Vector NTI Advance 10* (Invitrogen, USA).

### Culture conditions and RNA isolation

*M. hyopneumoniae* strain 7448 isolated from an infected swine (Embrapa, Santa Catarina, Brazil) [6] was grown in 25 ml of Friis broth [23] at 37°C for 24 h with gentle agitation in a roller drum. Total RNA was isolated with RNeasy Mini Kit (Qiagen, USA). For cell lyses, 0.7 ml of RNeasy Lysis Buffer (RLT buffer) in the presence of 0.134 M of β-mercaptoethanol was used per cultivation flask. The purification was performed according to the manufacturer’s instructions, with on-column DNaseI digestion using the RNase-Free DNase Set (Qiagen, Germany) and a second round of treatment with DNase I (Fermentas, USA). DNA absence was monitored to below PCR-detectable levels. Extracted RNA was analysed by gel electrophoresis and quantified in the Qubit system (Invitrogen, USA). Finally, RNA quality and integrity were determined by the evaluation of the RNA Integrity Number (RIN) using the Agilent 2100 Bioanalyzer (Agilent, USA). Values equal to or greater than 9.5 indicated sufficient quality.

### Reverse transcriptase PCR (RT-PCR) assay

For first-strand cDNA synthesis we used 1 μg of total RNA, 10 pmol of specific reverse primer (Additional file 1) and 10 mM of deoxynucleotide triphosphates. The mixture was heated to 70°C for 5 min and then incubated on ice for 5 min. First-strand buffer (Promega, USA), 0.1 M dithiothreitol, 40 U RNase inhibitor (Promega, USA) and 200 U M-MLV RT (Moloney Murine Leukemia Virus Reverse Transcriptase—Promega, USA) were then added to a total volume of 20 μl. The reaction was incubated at 37°C for 60 min followed by 15 min at 70°C for enzyme inactivation. A negative control was prepared in parallel, differing only by the absence of the RT enzyme.

PCRs included 1 U GoTaq DNA polymerase (Promega, USA), 5X of GoTaq buffer, 1 mM of each deoxynucleotide triphosphate, 10 pmol of each primer (Additional file 1) and 1 μl of the first-strand cDNA reaction in a final volume of 25 μl. A negative control of RT-PCR was prepared in parallel, which differed only by the absence of cDNA, and no genomic DNA was added to the reaction mixture for the PCR negative control. A PCR positive control was prepared using the genomic DNA of *M. hyopneumoniae* 7448 as the template. The PCR conditions were: 1 cycle at 94°C for 5 min followed by 30 cycles of 94°C for 30 s; denaturation and extension temperature and time varied according to each primer pair (Additional file 1). The final extension step was at 72°C for 10 min. Reaction products were analysed in 1.2% agarose gels.

### qPCR experimental design

Quantitative RT-PCR was performed using 1:50 cDNA prepared as described for RT-PCR as template and Platinum SYBR Green qPCR SuperMix-UDG (Invitrogen, USA) on the StepOne Real-Time PCR System (Applied Biosystems, USA). The qPCR reactions were carried out at 90°C for 2 min and 95°C for 10 min followed by 40 cycles of 95°C for 15 s and 55°C for 1 min each. The specificity of the synthesised products and the absence of primer dimers were visualised using a melting curve analysis for each reaction. Amplification efficiency for each primer pair was calculated using the LinRegPCR software application [24] and the mean efficiency values for each primer were added to Additional file 1. This efficiency value was used for the quantification analysis.

Relative expression of mRNA was calculated by the 2^-ΔCt^ method [25]. To control for all the experiments the threshold cycle (CT) values were normalised to the reference gene MHP7448_0333 [12,26]. The CT of each test target represents the average of three reactions. Three independent biological replicates were done for each target gene. We performed statistical analysis using GraphPad Prism 6 software. A two-tailed unpaired t-test was used to test for differences in the relative expression values between the regions before and after the terminator structure (P < 0.05).

## Results

### Prediction of terminator sequences

Computational analysis of *M. hyopneumoniae* genome using three different algorithms predicted 1068 terminators: 439 terminators using ARNold software, 334 using TransTermHP software and 295 using WebGesTer software (Table 1 and Additional file 2). These terminators were named using the initial letter of the software (a = ARNold, t = TransTermHP and w = WebGesTer) followed by a number. For example terminator t_001 is the first terminator predicted by TransTermHP software. Terminators named “wf” represent terminators classified as unstable terminators in the WebGesTer software (Additional files 2, 3 and 4).

**Table 1:** **Terminators predicted in** ***Mycoplasma hyopneumoniae***

	**Genic context**	**WebGesTer**	**TransTermHP**	**ARNold**	**Total**
Terminators predicted		295	334	439	1068
Class 1 terminator (t_c1_)	pC terminators	143	73	39	255
	mC terminators	40	20	5	65
	Internal terminators	95	99	42	236
	Total t_c1_	278	192	86	556

The classification criteria defined in Figure 1 were applied to validate the predicted terminators. Class 1 terminators, named t_c1_, should be positioned at the 3’ end of the CDS. Using this criterion, 556 terminators of the 1068 predicted terminators were selected (52%) (Table 1). The remaining predicted terminators were localised within coding regions or on the antisense strand and therefore, were not further analysed in this work.

The 556 t_c1_ terminators were analysed according to the genomic context of *M. hyopneumoniae*. The *M. hyopneumoniae* genome is organised in 33 monocistronic units (mCs) and 116 polycistronic units (pCs) containing two or more ORFs [9]. Therefore, systematic terminator localisation was performed in the downstream regions of all annotated ORFs in the genome. This analysis revealed the presence of 65 t_c1_ terminators at the end of mCs, 255 t_c1_ terminators at the end of pCs and 236 t_c1_ terminators at the 3’ end of some ORFs that were located internal in some polycistronic units (identified as internal genes) (Table 1). Moreover, more than one predicted terminator sequence can be found in some transcriptional units. Class 1 terminators were identified in all 33 mCs and in 106 of the 116 pCs (Additional files 3 and 4) suggesting that transcription termination occurs preferentially at the ends of mCs and pCs.

**Figure 3:**
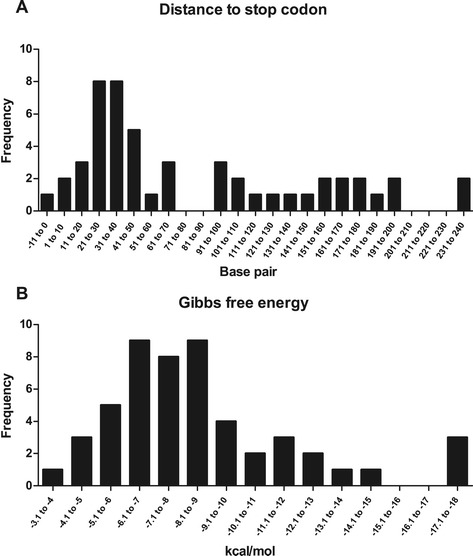
**Characteristics of class 2 terminators.** The graphics show the distribution of the class 2 terminators according to distance to stop codon **(A)** and the values of Gibbs free energy determined by the algorithm that predicted the terminator **(B)**. Negative distances indicate the overlap with the stop codon of the gene.

The class 1 terminators (t_c1_) were further divided into class 2, class 3 and class 4 terminators. To ensure the reliability of the generated data, the mC and pC t_c1_ terminators located at the same position by at least two algorithms were considered putative terminators and named class 2 terminators (t_c2_; Additional file 5). Using this approach we were able to identify class t_c2_ terminators in seven of the 33 mCs (21%) and in 39 of the 116 pCs (34%) (Table 2). Detailed analysis of the characteristics of these terminators revealed the presence of two distinguishing features: i) the distance between the start of the terminator and the stop codon of the target gene showed a range of −11 bp to 200 bp and ii) the values of Gibbs free energy (ΔG) were less than −4 kcal/mol (Figure 3). The presence of two class t_c2_ terminators at a distance greater than 230 bp from the stop codon (see Figure 3A) was identified at 3’ end of TU_09 (*fusA*) and TU_114 (MHP7448_r2). However, further analysis revealed the presence of other t_c2_ terminators at the 3’ end of these two pCs at position 39 or 41 for TU_09 and at positions 167, 185 or 186 for TU_114 (Additional file 2).

**Table 2:** **Terminator distribution according to the genome organisation of** ***M. hyopneumoniae***

**Class**	**mC**	**pC**
Class t_c2_	7 (21%)	39 (34%)
Class t_c3_	14 (43%)	36 (31%)
Class t_c4_	6 (18%)	11 (9%)
Without terminator	6 (18%)	30 (26%)
Total	33	116

To increase the number of mCs and pCs containing putative terminators (similar to class 2 terminators) all the remaining t_c1_ terminators were screened using the features defined in class 2 terminators (see Figure 3). Terminators that satisfied both criteria (distance to stop codon and ΔG) were reclassified as class 3 terminators (t_c3_) and those that displayed only one of the two criteria were named class 4 terminators (t_c4_). It is important to point out that, in contrast to class 2 terminators, the class 3 terminators were defined by only one algorithm. Class 3 terminators were identified in 14 of 33 mCs (43%) and in 36 of 116 pCs (31%) increasing the number of mCs and pCs with putative terminators to 21 (64%) and 75 (65%), respectively (Table 2). Class 4 terminators (t_c4_) were found in six mCs (18%) and eleven pCs (9%) of the *M. hyopneumoniae* genome.

### Experimental validation of M. hyopneumoniae terminators

RT-PCR and qPCR were used to test whether the predicted terminators were related to transcription termination in *M. hyopneumoniae*. The terminator validation hypothesis was based on the following premises: i) mC or pC transcripts ended at the predicted terminator sequences; and ii) no mC or pC transcripts were present after the predicted terminator sequences. Primer pairs (Figure 2) were positioned to validate this hypothesis, similar to the terminator validation previously used by Arrebola et al. [27].

**Figure 4:**
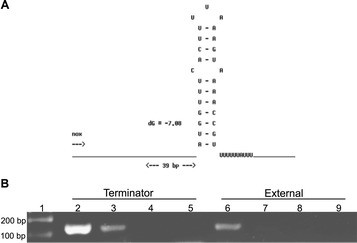
**Experimental analyses of polycistronic unit 10 (TU_10) by RT-PCR. A**: Structure and characteristics of class 2 terminator w_014, predicted by the software WebGesTer. **B**: agarose gel electrophoresis of RT-PCR products obtained from primers used to analyse expression in TU_10. The reactions were performed in the presence (+) and absence (−) of reverse transcriptase. Lanes 2, 3, 4 and 5 represent the reaction with the primer positioned upstream of the terminator. Lanes 6, 7, 8 and 9 represent the reaction with the primer positioned downstream of the terminator. Results of the reaction with positive cDNA are presented in lanes 3 and 7. Control reactions (DNA) using genomic DNA as the template are presented in lanes 2 and 6. Lanes 4 and 8 represent the control reaction of cDNA synthesis while lanes 5 and 9 are the control of the PCR reaction. Lane 1 contains a 100 bp ladder.

RT-PCR was performed for 15 putative terminator sequences. Among the putative terminator sequences analysed, four belonged to class t_c2_, one to class t_c3_ and 10 to class t_c4_ (Table 3). The Figure 4 shows the validation of a t_c2_ terminator (w_014) in TU_10, used as example, where two primer pairs were designed for RT-PCR analysis. As shown in Figure 2, primer pairs were positioned to analyse the presence of transcripts upstream and immediately downstream of the predicted terminator. TU_10 is composed of genes MHP7448_0081 and *nox*. At the end of the *nox* gene, three t_c2_ terminators were identified (Additional file 2). The experimental analysis considered the w_014 terminator located at 39 bp of the *nox* gene stop codon. This terminator is formed of a stem of 11 nucleotides with only one mismatch, one loop of three nucleotides, the U-tract and has a ΔG of −7.08 kcal/mol (Figure 4A). As demonstrated in Figure 4B (lines 3 and 7) amplification was observed only when primers were positioned upstream of the terminator sequence indicating that the w_014 terminator is responsible for transcription termination in *M. hyopneumoniae* TU_10.

**Table 3:** **Predicted terminators analysed by RT-PCR**

**Terminator**	**Validation** ^**1**^	**Localisation**	**Class**	**Gene** ^**2**^	**Distance (bp)** ^**3**^	**ΔG** ^**4**^
w_053	No	mC_15	t_c3_	*pyrG*	82	−5.01
wf_491	TE	mC_28	t_c4_	*pdhD-1*	193	−4.51
wf_204	No	mC_30	t_c4_	*pulA*	131	−2.48
w_202	No	mC_31	t_c4_	*dam*	195	−4.69
wf_513	TE	mC_33	t_c4_	*gcp*	190	−4.36
t_020	TE	TU_010	t_c2_	*nox*	40	−6.5
wf_075	No	TU_036	t_c4_	*rpsT*	−9	−3.94
w_147	No	TU_039	t_c4_	*lip*	181	−4.76
wf_375	TE	TU_060	t_c4_	*glpF*	29	−2.22
wf_137	TE	TU_066	t_c4_	*metG*	149	−3.21
w_076	No	TU_085	t_c2_	*pdhD*	37	−9.01
t_264	TE	TU_089	t_c2_	*tuf*	34	−7.5
wf_202	TE	TU_098	t_c4_	*fpg*	52	−2.53
wf_215	TE	TU_103	t_c4_	MHP7448_0621	198	−3.11
w_102	TE	TU_113	t_c2_	MHP7448_0665	43	−11

^1^Presence of functional activity. TE — transcription termination; TD — transcription decreasing; No — without difference in transcript upstream and downstream of the terminator sequence.

^2^Gene at the end of the transcription unit.

^3^Distance between stop codon of the gene and the first nucleotide of terminator sequence.

^4^Value of free energy of Gibbs identified by the software that predicted the terminator.

Transcription termination was also demonstrated in the presence of t_c2_ terminators t_264 and w_102 located at TU_89 and TU_113, respectively (Table 3). In six t_c4_ terminators (wf_491, wf_513, wf_375, wf_137, wf_202 and wf_215), amplification product was detected only when using primers located upstream of the putative terminator sequence, suggesting that the presence of t_c4_ terminators at the 3’ end of mC_28, mC_33, TU_60, TU_66, TU_98 and TU_103 were responsible for transcription termination (Table 3). These data shows that the putative intrinsic terminators are functional in mycoplasmas.

In the other class 2 terminator (w_076), class 3 terminator (w_053) and four class 4 terminators (wf_204, w_202, wf_075, w_147), amplification products were detected when primers located downstream of the terminator were used and, therefore, could not be validated by RT-PCR. However, it is possible that some terminators, although incapable of efficient transcription termination, could cause a decrease in the level of transcription. To confirm this hypothesis, RNA expression level was quantified using qPCR.

**Figure 5:**
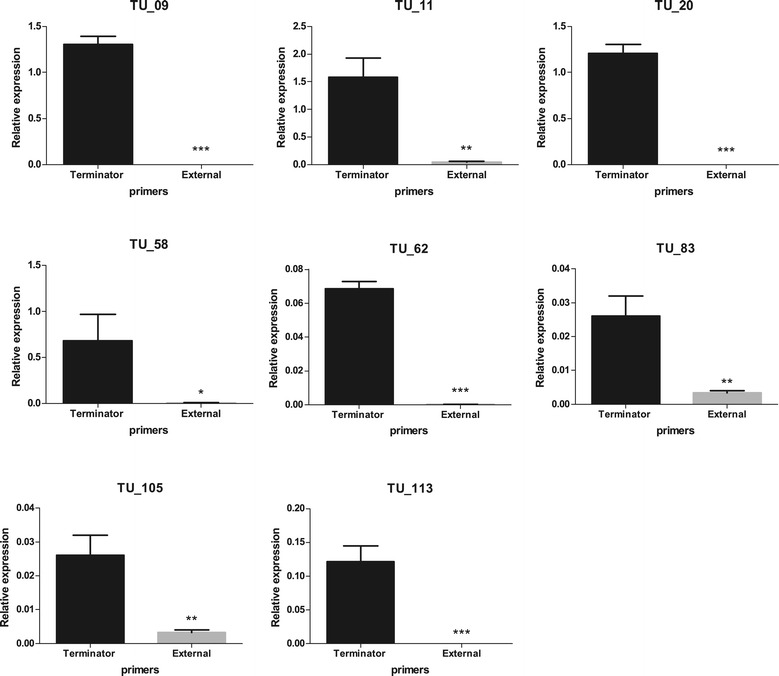
**Analysis of relative expression by qPCR of class 2 terminators.** Graphics represent the analysis of the terminator present in each of the pCs (terminator w_109 in TU_09, terminator t_022 in TU_11, terminator t_055 in TU_20, terminator a_207 in TU_58, terminator t_173 in TU_62, terminator a_310 in TU_83, terminator w_093 in TU_105 and terminator w_102 in TU_113). Column “Terminator” shows the level of expression of the primer positioned upstream of the terminator and the column “Extern” represents the data for the primer positioned downstream of the terminator. Data are presented as mean ± standard deviation of three independent experiments. Asterisks indicate statistically significant differences in levels of expression downstream of the terminator; *0.01 < P < 0.05; **0.001 < P <0.01; ***P < 0.001.

**Figure 6:**
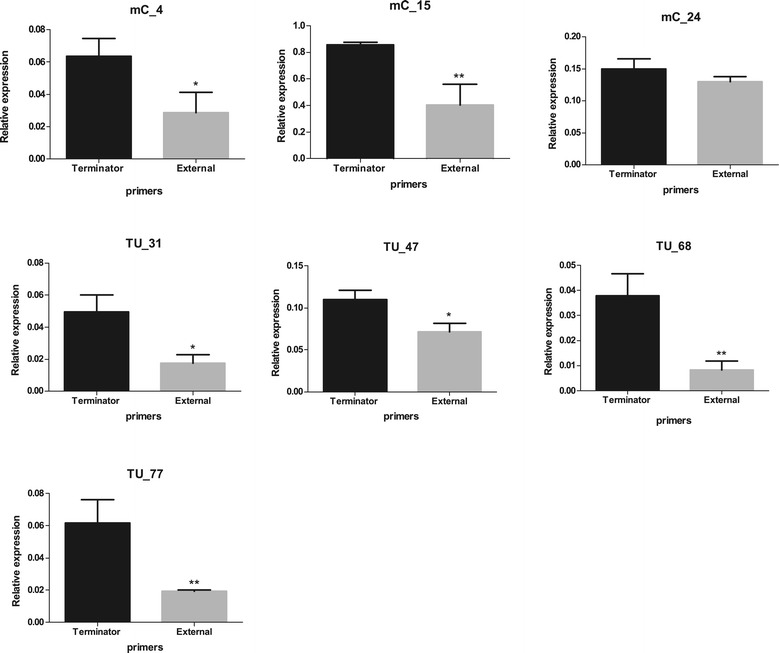
**Analysis of relative expression by qPCR of class 3 terminators.** Graphic represent the analysis of terminator present in mCs and pCs (w_016 in mC_04, w_053 in mC_15, w_082 in mC_24, w_140 in TU_31, w_055 in TU_47, wf_140 in TU_68 and w_181 in TU_77). Column “Terminator” shows the level of expression of the primer positioned upstream of the terminator and the column “Extern” represents the data of the primer positioned downstream of the terminator. Data are presented as mean ± standard deviation of three independent experiments. Asterisks indicate statistically significant differences in expression levels downstream of the terminator; *0.01 < P < 0.05; **0.001 < P < 0.01.

**Figure 7:**
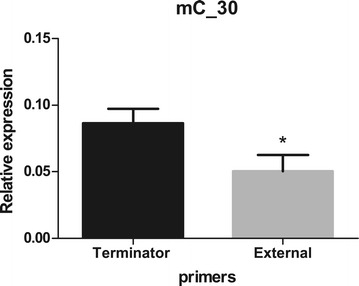
**Analysis of relative expression by qPCR of class 4 terminator.** Graphic represents the analysis of the terminator wf_204 present in mC_30. Column “Terminator” shows the level of expression of the primer positioned upstream of the terminator and the column “Extern” represent the data of the primer positioned downstream of the terminator. Data are presented as means ± standard deviation of three independent experiments. Asterisk indicates a statistically significant difference in expression levels downstream of the terminator wf_204 in mC_30; *P < 0.05.

Real-time quantitative PCR was performed for selected class 2, class 3 and class 4 terminators based on the feasibility of primer design, as the *M. hyopneumoniae* genome has an intergenic low GC content. A total of 16 terminators were analysed by qPCR (Table 4): eight t_c2_ (Figure 5), seven t_c3_ (Figure 6) and one t_c4_ terminators (Figure 7). In order to compare RT-PCR and qPCR methodologies, terminator w_102 of TU_113, a t_c2_ terminator previously confirmed by RT-PCR (Table 3), was also analysed by qPCR (Figure 5).

**Table 4:** **Predicted terminators analysed by qPCR**

**Terminator**	**Validation** ^**1**^	**Localisation**	**Class**	**Gene** ^**2**^	**Distance (bp)** ^**3**^	**ΔG** ^**4**^
w_016	TD	mC_04	t_c3_	*rbgA*	88	−6.06
w_053	TD	mC_15	t_c3_	*pyrG*	82	−5.01
w_082	No	mC_24	t_c3_	*gyrA*	75	−6.41
wf_204	TD	mC_30	t_c4_	*pulA*	131	−2.48
w_109	TE	TU_009	t_c2_	*fusA*	39	−17.8
t_022	TD	TU_011	t_c2_	*deoA*	119	−10
t_055	TE	TU_020	t_c2_	*rpmB*	49	−7.6
w_140	TD	TU_031	t_c3_	*trmE*	35	−7.81
w_055	TD	TU_047	t_c3_	*ugpQ*	18	−8.9
a_207	TD	TU_058	t_c2_	*hit*	27	−5.8
t_173	TD	TU_062	t_c2_	MHP7448_0372	43	−9.4
wf_140	TD	TU_068	t_c3_	*ruvB*	42	−3.91
w_181	TD	TU_077	t_c3_	*rplA*	107	−6.19
a_310	TD	TU_083	t_c2_	MHP7448_0494	24	−5.12
w_093	TD	TU_105	t_c2_	MHP7448_0628	109	−8.35
w_102	TE	TU_113	t_c2_	MHP7448_0665	43	−11

^1^Presence of functional activity. TE – transcription termination; TD – transcription decreasing; No – without difference in transcript upstream and downstream of the terminator sequence.

^2^Gene at the end of transcription unit.

^3^Distance between stop codon of the gene and the first nucleotide of terminator sequence.

^4^Value of free energy of Gibbs identified by the software that predicted the terminator.

For the majority of tested terminators primer efficiency was greater than 80%. Only the primers used to analyse the terminator in TU_62 showed, in both primer pairs, a lower efficiency of 65% (Additional file 1). Moreover, the primer pairs designed for terminator sequences w_102 in TU_113 and t_055 in TU_20 revealed differences in efficiency between upstream and downstream primers of around 50% (Additional file 1). This result is probably related to the absence of detected transcripts in the qPCR (Figure 5).

The eight class 2 transcriptional terminators analysed by qPCR were localised at 3’ end of the last gene in TU_09, TU_11, TU_20, TU_58, TU_62, TU_83, TU_115 and TU_113 (Figure 5). Real-time quantitative PCR demonstrated that only transcripts found for TU_09, TU_11, TU_20, TU_58 and TU_113 had similar expression levels to the gene used for normalization (MHP7448_333) in these experiments. The transcriptional terminators were characterised by comparison between transcripts level using primers upstream of the putative terminator sequence (Terminator) and downstream of the terminator sequence (External). Terminators w_109 in TU_09, t_055 in TU_20 and w_102 in TU_113 can be considered valid terminators as no transcripts were detected downstream of the terminator sequence (Figure 5 see External). The other t_c2_ class terminators, t_022 in TU_11, a_207 in TU_58, t_173 in TU_62, a_310 in TU_83, and w_093 in TU_105 displayed a significant (0.001 < P < 0.05) decrease in transcription when downstream primers were used (see Figure 5 Terminator versus External) and also could be related to transcriptional termination.

Transcriptional terminators classified as class 3 (w_016 in mC_04, w_053 in mC_15, w_082 in mC_24, w_140 in TU_31, w_055 in TU_47, wf_140 in TU_68 and w_181 in TU_77) and class 4 (wf_204 in mC_30) terminators were also analysed by qPCR (Figure 6 and Figure 7). Similar to some t_c2_ class terminators, all t_c3_ and t_c4_ class terminators displayed a significant (0.01 < P < 0.05) decrease in transcription when downstream primers were used, with exception of w_082 in mC_24 (Figure 6 and Figure 7; see Terminator versus External). Interestingly, a second t_c3_ (w_083) putative terminator was localised at 74 bp downstream of the first terminator w_082 (Additional file 2). The presence of two putative terminators in this monocistronic unit suggests that they could have complementary functions.

**Figure 8:**
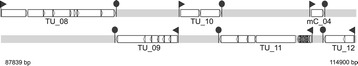
***M. hyopneumoniae*** **genome organisation containing promoter and terminator transcription sites.** Figure shows the transcription unit organisation in the genome region from 87,839 to 114,900 base pair. Genome organisation and promoter identification were previously defined [9,11]. Black arrows represent promoter sequence and black circle represent terminator sequence.

Aiming to summarize our results Figure 8 shows the localisation of both promoter sequences and intrinsic terminators according the genome organisation of *M. hyopneumoniae*. Previous works of our group have demonstrated that the genome is organized preferably in long transcriptional units containing promoter sequences upstream the start codon of the first gene of the units [9,11]. Among the putative class 2 and class 3 terminators, represented in Figure 8, the functional terminators at the end of TU_09, TU_10, TU_11 and mC_04, were also experimentally demonstrated by RT-PCR and qPCR analyses (Tables 3 and 4).

## Discussion

The importance of the characterisation of Rho-independent termination in mycoplasma can be related with the accurate prediction of transcription units in these bacteria. In general, the properties of intrinsic terminators, such as sequence and the structural features of the hairpin and, in some cases, the U-tract of the nascent transcript, could be involved in transcription termination. Previous works have demonstrated the predominance and conservation of Rho-independent or intrinsic termination among the Mollicutes class [3,4,16].

We identified transcription terminators for *M. hyopneumoniae in silico* using three algorithms that demonstrated differences in efficiency of prediction. This method allowed the prediction of 1068 terminators in *M. hyopneumoniae* genome. Moreover, detailed analysis revealed the prediction of at least one terminator sequence in 253 of the 705 *M. hyopneumoniae* ORFs (35.9%).

In comparison to *E. coli*, the intrinsic terminators found in the *M. hyopneumoniae* genome display relatively high values of Gibbs free energy density of stem-loop formation correlated to the low content of guanine and cytosine in the Mollicutes genome [13]. Similar values were also found in intrinsic terminators of *M. genitalium* and *M. pneumoniae* and are probably responsible for previous difficulties identifying intrinsic terminators in *M. pneumoniae* based on analysis of average RNA folding energy near stop codons [3,14]. The presence of a U-tract following the stem-loop structure was detected for the majority of the predicted *M. hyopneumoniae* terminators. However, approximately 20% of *M. hyopneumoniae* stem-loop formation revealed absence of the U-tract tail of the nascent transcript. These results are similar to those found in other mycoplasma species, suggesting that the U-tract tail is not an essential requisite for intrinsic terminators in this genus [4]. Nevertheless, the efficiency of transcription termination is maintained in intrinsic terminators without the U-tract [28,29].

Detailed localisation analysis of the 1068 predicted terminators allowed us to select only those found at the 3’ end of pCs or mCs. Using this approach we were able to distinguish inter- from intracistronic terminator-like structures and select 320 putative Rho-independent terminators at the end of mCs and pCs. The prediction efficiency was different among the software, reflecting the specific algorithm used for each one. The best results were obtained with the software WebGesTer, as is the only one of the three software packages that takes into consideration the gene context, considering a region ranging from −20 to +270 bp from the stop codon of CDS, and allows the detection of terminators without a U-tract [17]. Of the 295 terminators predicted by this program 279 were classified as class t_c1_. The software TransTermHP identified 191 terminators class t_c1_. The lower number of predicted terminators found by the TransTermHP software is related to the restrict search of palindromic sequences in regions with the presence of at least three thymines in sequence. Therefore, the TransTermHP program is able to found only canonical sequences [16]. The ARNold software use the patterns defined for *E. coli* and *B.subtilis* and do not take in consideration the gene context in which the terminator occur [21]. This probably explains why only 86 of the 439 (19%) predicted terminators, classified as class 1 terminators, were found by this program. The remaining terminators predicted by ARNold program were localised within coding regions or on the antisense strand and therefore were not analysed in this paper.

Class t_c1_ terminators were identified in all 33 mCs and in 106 of 116 pCs. This method was unable to localise terminators in only 10 (8.6%) pCs. Interestingly, in seven of them (TU_15, TU_16, TU_52, TU_72, TU_78, TU_104 and TU_106) the last ORF is classified as hypothetical. Moreover, terminator sequences were found upstream of the last ORF in TU_15, TU_16, TU_72 and TU_106 suggesting that transcription could be terminated before the last ORF in these polycistronic units. In three pCs (TU_72, TU_104 and TU_115) the presence of a gene similar to one that codes for transposase was detected. Therefore, we suggest that Rho-independent termination occurs at specific sequences in *M. hyopneumoniae* genome.

Aiming to distinguish *M. hyopneumoniae* Rho-independent terminators from random stem-loop structure sequences we established rules to discover their distinguishing properties. We used class 2 terminator classification (t_c2_) to propose a model of intrinsic terminators and as a decision rule to define as putative terminators in *M. hyopneumoniae* genome. The decision rule had four parameters: i) an inverted repeat in the primary DNA sequence positioned at the 3’ end of pC or mC followed or not by a short stretch of thymine residues; ii) predicted by at least two different algorithms; iii) distance between the terminator first nucleotide and the stop codon of the target gene range from −11 to 200 bp; and iv) the values of Gibbs free energy (ΔG) should be less than −4 kcal/mol. However, the application of these four rules validated terminators *in silico* in only 21% of the mCs (seven of the 33) and in 34% of the pCs (39 of the 116).

Therefore, to increase the number of putative terminators of the 320 class 1 terminators we lowered the specificity of the class 2 decision rule as follows: class t_c3_ — class 1 intrinsic terminators validated by only one algorithm, maintaining rules i, iii and iv, described above; and class t_c4_ — class 1 intrinsic terminators validated by only one algorithm, maintaining rules i and iii or iv. Using these new rules the numbers of transcriptional units containing putative terminators analysed *in silico* increased to 27 mCs and 86 pCs (t_c2_, t_c3_ and t_c4_). Therefore, putative intrinsic terminators have been found, by *in silico* analysis, in 76% of transcription units of the *M. hyopneumoniae* genome (82% of the 33 mCs and in 74% of the 116 pCs).

Previous studies have demonstrated the presence of intrinsic terminators in genomes of *M. genitalium* (20%) and *M. pneumoniae* (24%) [16], and also in specific genes such as the 16S-23S rRNA operon of *M. hyopneumoniae* [30], the P1 operon of *M. genitalium* [31], the MgPa and P65 operons, and the *ldh* gene of *M. pneumoniae* [32-34]. Moreover, transcriptional terminators in bacterial genomes belonging to the Firmicutes phylum have also been predicted revealing a high level of conservation [3].

Little information on transcription termination in *M. hyopneumoniae* is available. To validate the *in silico* putative terminators, experiments were performed for analysis of the transcription end guided by putative terminators. The experimental analyses evaluated 28 of the 113 putative intrinsic terminators belonging to class 2, class 3 and class 4 terminators. The number of terminators experimentally analysed (24%) was limited due to difficulties in designing primers for some locations as these intergenic regions are characterised by a high adenine and thymine content. In eleven putative terminator sequences, located at the 3’ end of two mCs and nine pCs, RT-PCR or qPCR demonstrated transcription termination. However, for some intrinsic terminators (12 terminators) transcription continued through the terminator sequence but the level of transcription decreased. The degree of transcriptional change that occurs during quantitative PCR suggests that RNA polymerase continues past the terminator sequence and gradually release the RNA, as demonstrated by transcriptome analysis [10]. As the termination efficiency of an intrinsic terminator is directly related to the stability of the stem-loop structure [4] the relatively high ΔG values in intrinsic terminators could be responsible for the read-through of some *M. hyopneumoniae* terminator sequences. Supporting our findings, the presence of functional intrinsic terminators was previously demonstrated for genes in other mycoplasma species such as *hmw* of *M. pneumoniae*, *vmpaU* of *Mycoplasma agalactiae* and for the operon *ftsZ* of *M. genitalium* [18-20].

## Conclusion

*In silico* predictions, combined with experimental analysis, confirmed the presence of intrinsic terminators in *M. hyopneumoniae* genome. The Figure 8 summarizes the current knowledge related to transcription in *M. hyopneumoniae*. The localisation of terminators sequences at the 3’ end of mCs and pCs supports previous findings that the *M. hyopneumoniae* genome is organised preferentially in TUs containing two or more genes [9,10]. Moreover, the experimental results suggest that at least some terminators have a functional role in mycoplasma. Therefore, the presence of terminator sequences associated with the identification of promoter sequences in *M. hyopneumoniae* transcriptional units (mC and pCs) [12] suggest that transcription runs on from an upstream promoter and terminates at specific stem-loop regions.

## Additional files

Additional file 1:
**List of primers used in RT-PCR and qPCR, showing the sequence, melting point and product size of the primers pairs used in RT-PCR and qPCR.** Amplification efficiency was calculated by LinRegPCR for primers used in qPCR. Additional file 2:
**Classes 1, 2, 3 and 4 putative terminators predicted in** ***M. hyopneumoniae*** **.** Characteristics of predicted terminators in the region 3’ end after the coding sequence. These terminators were classified in classes 1, 2, 3 and 4 according to the criteria described in the text. Additional file 3:
**Mapping of terminators predicted in monocistronic units (mC) of** ***M. hyopneumoniae*** **.** Presence of terminators class 1 in monocistronic units according the genome organisation of *M. hyopneumoniae*. Additional file 4:
**Mapping of terminators at the end of polycistronic units and in internal genes of** ***M. hyopneumoniae*** **genome.** Presence of terminators class 1 in polycistronic units according the genome organisation of *M. hyopneumoniae*. Additional file 5:
**Definition of class 2 terminators.** The scheme represents a region of 101600 at 111600 bp in *M. hyopneumoniae* genome with the polycistronic units 10 (*nox*) and 11 (*deaA*). TU_10 showed three predicted terminators in the same position; two were predicted by TransTermHP (red) and one by WebGesTer (yellow). TU_11 has three predicted terminators in the same position that were predicted by all three software algorithms. The terminator in orange was predicted by ARNold.

## Abbreviations

IR: Intergenic region
CDS: Coding sequences
ORF: Open reading frame
t_c1_: class 1 terminator
t_c2_: class 2 terminator
t_c3_: class 3 terminator
t_c4_: class 4 terminator
ΔG: Gibbs free energy
RT-PCR: Reverse transcriptase PCR
qPCR: quantitative real time PCR
TU: Transcription unit
pC: polycistronic unit
mC: monocistronic unit
CT: Threshold cycle
