# Supplementary figures and images for: Intrinsic terminators in Mycoplasma hyopneumoniae transcription
Source: BMC Genomics. 2015 Apr 8;16(1):273. doi: 10.1186/s12864-015-1468-6 (PMC4411717; doi:10.1186/s12864-015-1468-6)

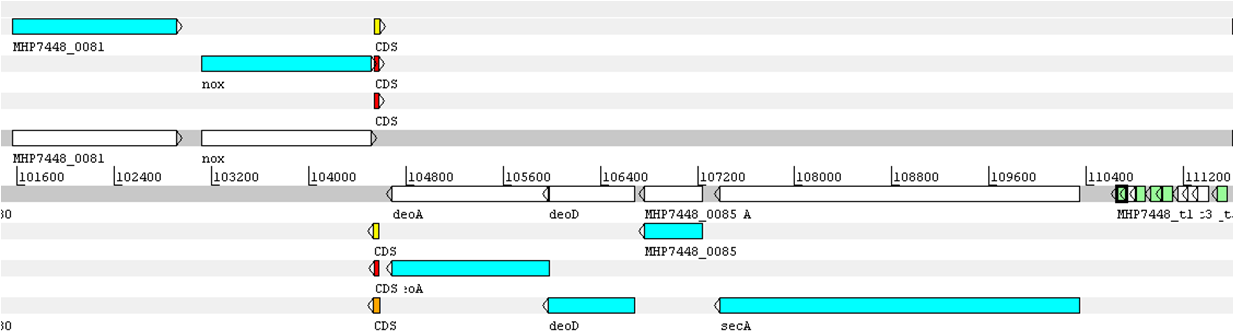

Supplement: Additional file 5: — Definition of class 2 terminators. The scheme represents a region of 101600 at 111600 bp in M. hyopneumoniae genome with the polycistronic units 10 (nox) and 11 (deaA). TU_10 showed three predicted terminators in the same position; two were predicted by TransTermHP (red) and one by WebGesTer (yellow). TU_11 has three predicted terminators in the same position that were predicted by all three software algorithms. The terminator in orange was predicted by ARNold. [file 12864_2015_1468_MOESM5_ESM.tiff]
